# Implementation of Paediatric Life Support Recommendations: A Survey in the D-A-CH Region

**DOI:** 10.3390/children13080986

**Published:** 2026-07-24

**Authors:** Franziska Markel, Bettina Lück, Michael Sasse, Philipp Jung, Florian Hoffmann, Ellen Heimberg, Martin Olivieri, Sebastian Brenner, Bernd Landsleitner, Francesco Cardona, Eva Maria Jordi-Ritz, Benjamin W. Ackermann

**Affiliations:** 1 Department of Psychocardiology, Department of Developmental Pediatrics, Deutsches Herzzentrum der Charité (DHZC), Charité–Universitätsmedizin Berlin, 13353 Berlin, Germany; melanie-franziska.markel@charite.de; 2 Department of Congenital Heart Disease–Pediatric Cardiology, Deutsches Herzzentrum der Charité (DHZC), Charité–Universitätsmedizin Berlin, 13353 Berlin, Germany; 3 Charité–Universitätsmedizin Berlin, Corporate Member of Freie Universität Berlin and Humboldt-Universität zu Berlin, 10117 Berlin, Germany; 4 Division of Neonatology, Department of Women’s and Children’s Medicine, Medical Faculty, Leipzig University, 04103 Leipzig, Germany; 5 Department of Paediatric Cardiology and Intensive Care Medicine, Hannover Medical School (MHH), 30625 Hannover, Germany; 6 Department of Paediatrics, Staedtisches Klinikum Lueneburg, 21339 Lueneburg, Germany; 7 Department of Pediatric Intensive and Emergency Care, Dr. Von Hauner Children’s Hospital, LMU University Hospital, LMU Munich, 80337 Munich, Germany; 8 Department of Paediatric and Adolescent Medicine, Klinikum Dritter Orden München-Nymphenburg, 80638 Munich, Germany; 9 Department of Paediatric Cardiology, Pulmonology and Intensive Care Medicine, University Children’s Hospital Tuebingen, 72076 Tuebingen, Germany; 10 Department of Pediatrics, Division of Pediatric Emergency and Intensive Care Medicine, University Hospital Carl Gustav Carus Dresden, Technische Universität Dresden, 01307 Dresden, Germany; 11 Department of Anesthesia and Intensive Care Medicine, Cnopf Chidren’s Hospital, Hospital Hallerwiese, 90419 Nuremberg, Germany; 12 Division of Neonatology, Pediatric Intensive Care and Neuropediatrics, Department of Pediatrics and Adolescent Medicine, Medical University of Vienna, 1090 Vienna, Austria; 13 Department of Paediatric Anaesthesia, University Children’s Hospital Basel (UKBB), 4056 Basel, Switzerland

**Keywords:** paediatric cardiac arrest, resuscitation training, resuscitation guidelines, patient safety, implementation

## Abstract

**Highlights:**

**What are the main findings?**

- Structural prerequisites for paediatric resuscitation—including Paediatric Early Warning Systems, designated resuscitation supervisors, structured debriefings, and post-resuscitation care guidelines—remain markedly underimplemented across paediatric hospitals in Germany, Austria, and Switzerland, with fewer than 1% of respondents working in fully compliant settings.
- No significant differences in implementation rates were observed by hospital size or type, indicating that barriers are systemic rather than institution-specific and affect the entire D-A-CH region equally.

**What are the implications of the main findings?**

- Mandatory integration of certified Paediatric Advanced Life Support training into continuing medical education requirements, combined with employer-monitored compliance, is urgently needed to close the implementation gap and reduce preventable neurological harm and death in children following cardiac arrest.
- Supranational coordination across Germany, Austria, and Switzerland is essential to harmonise training standards and structural requirements, as the identified deficiencies cannot be addressed by individual institutions alone.

**Abstract:**

Background: Implementing recommendations to improve in-hospital resuscitation is a complex process. The extent to which an advisory statement formulating ten theses to improve resuscitation quality in paediatrics is applied in clinical practice across Germany, Austria, and Switzerland (D-A-CH-region) remains unclear. Methods: A web-based cross-sectional survey was conducted among paediatric physicians in the D-A-CH region between November 2022 and May 2023, comprising 50 questions focusing on experience and safety in paediatric emergency management and on the structural conditions at hospitals required to implement the ten theses. Ethics approval was obtained from the Ethics Committee Leipzig, Germany (218/19-ek). Results: Structural recommendations were implemented to varying and often limited degrees: An emergency telephone chain was available in 91% of workplaces, 19% had a Paediatric Early Warning System, and 38% had a designated resuscitation supervisor. Although in-house training was available to 89% of respondents, 31% had not participated in in-house training in the preceding 12 months, and 37% had not attended a certified resuscitation course in the last five years. A total of 48% of respondents reported that structured debriefings following emergency events were rarely or never conducted. Internal guidelines for post-resuscitation care were available in 26% of institutions. Only seven respondents (less than 1%) worked in a setting where all the recommendations surveyed were in place. No significant differences were observed according to hospital size or type. Conclusions: Structural implementation of paediatric resuscitation recommendations remains markedly inadequate across the D-A-CH region, with likely multifactorial causes that are not yet fully understood.

## 1. Introduction

The European Resuscitation Council (ERC) has recently published updated paediatric life support (PLS) guidelines. Beyond incremental adjustments, a new focus has been placed on post-resuscitation care, neuroprognostication, and, for the first time, on care following hospital discharge [1]. It is increasingly evident that patient outcomes are determined not only by the resuscitation process itself, but by the effective functioning of the entire chain of survival.

The chain of survival comprises a complex sequence of components, including prevention of cardiac arrest, effective Paediatric Basic Life Support (PBLS) and Paediatric Advanced Life Support (PALS), as well as post-resuscitation treatment. Sustaining each component demands not only human, financial, and temporal resources, but also institutional awareness and a deeply embedded training culture.

The incidence of in-hospital cardiac arrest (IHCA) in children in the D-A-CH-region remains unknown. International data suggest that the incidence of paediatric IHCA is highest in infants at approximately 70 per 100,000, decreasing with age to around 5 per 100,000 in adolescence [2,3]. Across studies, survival to discharge after paediatric IHCA ranges from 30% to 50% with approximately 47% of survivors experiencing a favourable neurological outcome [4]. A recent European survey of paediatric cardiac arrest registries further highlighted substantial heterogeneity in reported incidence, survival, and neurological outcomes across centres and countries [5]. Due to the low prevalence of cardiac arrest in children, resuscitation is only rarely required, and emergency teams are frequently assembled on an ad hoc basis without established team structures [2,5,6]. High-quality resuscitation performance under these conditions is difficult to achieve and maintain. Numerous simulation and manikin studies have demonstrated that high-quality training is essential to performing this task effectively [7]. Although no consensus exists regarding optimal training frequency, the Education, Implementation, and Teams (EIT) Task Force of the International Liaison Committee on Resuscitation (ILCOR) recommends spaced learning with regular repetition, rather than massed learning at single time points, to maintain team competence [8].

**Figure 1:**
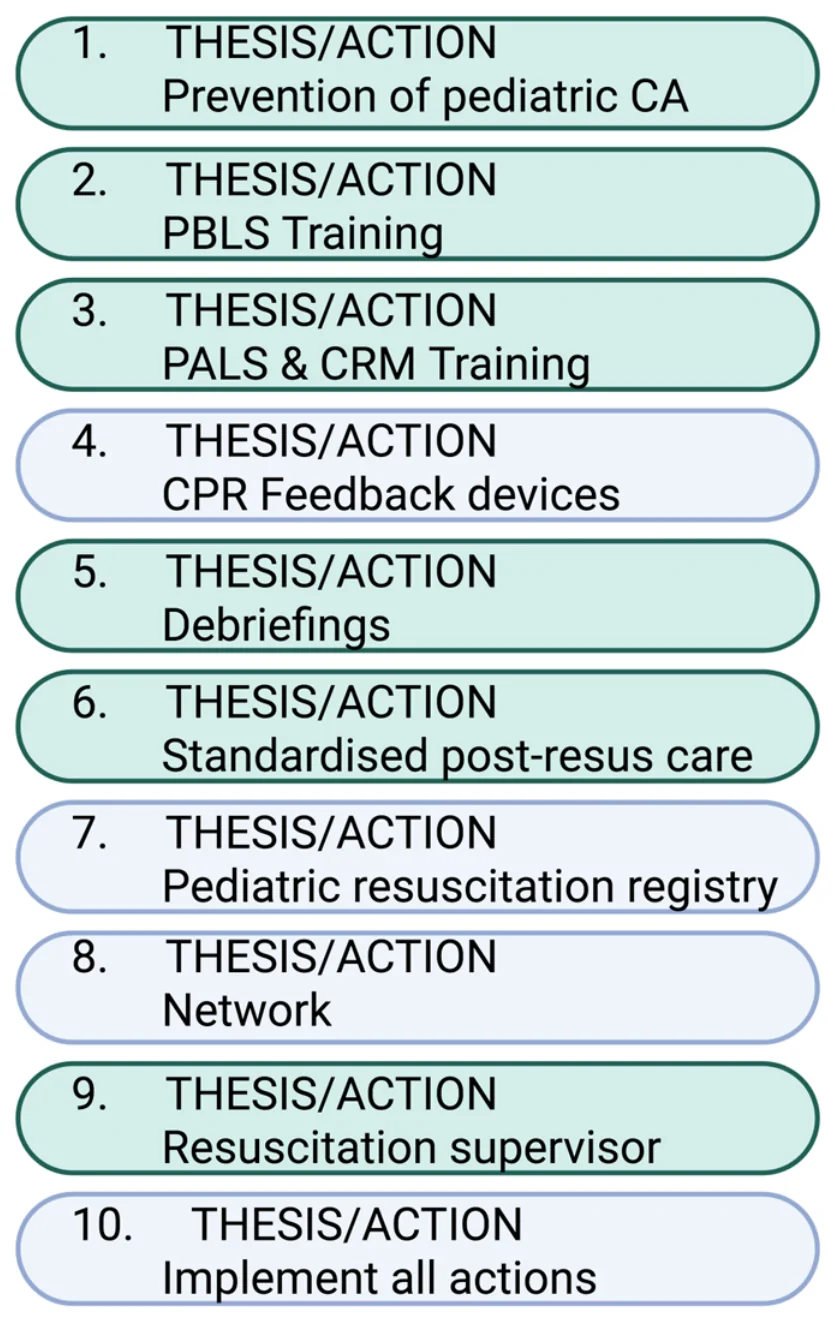
Ten theses/action plans to improve quality of paediatric cardiopulmonary resuscitation. Resus = resuscitation. The implementation of the theses in the green boxes are included in this survey [9].

Nevertheless, the extent to which paediatric teams across Germany, Austria, and Switzerland have implemented resuscitation guidelines remains unclear. Jung et al. have translated the requirements arising from existing guidelines into ten actionable theses for clinical teams (Figure 1) [9]. These action plans address the full chain of survival: prevention of deterioration through structured early-warning systems, high-quality PBLS and PALS training, team trainer skills, post-event debriefing, and structured post-resuscitation care including clear goals for the post-resuscitation phase, representing a practical framework for institutional self-assessment and quality improvement. In collaboration with the Paediatric Life Support working group of the GRC, a nationwide survey was conducted to assess the implementation of these theses and to evaluate respondents’ self-reported experience and confidence in paediatric emergency care.

## 2. Materials and Methods

### 2.1. Study Design

This study was designed as a nationwide cross-sectional survey among medical professionals in Germany, Austria, and Switzerland. The survey comprised 50 questions structured into three sections: (1) demographic data; (2) personal experience, self-assessed safety in paediatric emergency management, and training preferences; and (3) structural conditions for implementing paediatric emergency care at the respondents’ hospitals (Supplementary Material File S1: Study Questionnaire).

The survey was designed by the author group and refined across two consensus meetings. Six of the ten theses proposed by Jung et al. were included (Theses 1, 2, 3, 5, 6, and 9); the remaining four were excluded for different reasons (Theses 4, 7, and 8 were omitted because these items concern structural and administrative features that residents early in training may be unable to report reliably: Thesis 4 [objective and subjective CPR-feedback systems], Thesis 7 [obligatory participation in a resuscitation registry], and Thesis 8 [structured inter-hospital networking]. Omitting them also shortened the questionnaire and protected the participation rate. Thesis 10 [implementation of all recommendations] was covered by the other items) (Figure 1). The instrument was piloted by five individuals with academic qualifications from diverse professional backgrounds (medical and non-medical) and subsequently adapted. As this instrument was designed to capture structural implementation features rather than a latent psychometric construct, no formal psychometric validation was performed.

The final version was integrated into the LimeSurvey platform (version 5, LimeSurvey GmbH, Hamburg, Germany). The present paper focuses on the organisational and structural implementation of the individual theses; empirical findings on self-confidence and personal experience will be reported in a separate publication.

### 2.2. Data Collection

A non-random, multi-stage, gatekeeper-based sampling approach was employed. All paediatric clinics in Germany authorised for further training in paediatric and adolescent medicine were identified. Invitations were sent to the 408 heads of paediatric departments (chief physicians) at 327 paediatric hospitals in Germany, with a request to forward the survey link to physicians in paediatric specialty training within their departments. The survey was additionally advertised in two national paediatric journals and at professional training events, encouraging voluntary participation. In Austria, the survey link was sent to the relevant children’s hospitals via the paediatric medical association. This approach constitutes a gatekeeper-based and snowball-like sampling strategy.

Inclusion and exclusion criteria: The analysis was restricted to respondents identifying themselves as physicians. Non-physician staff or respondents who did not indicate their professional status were excluded in the analysis.

Consent and data protection: Participation was entirely voluntary and anonymous, no server data or identifying information were recorded. Informed consent was obtained after a consent checkbox has been actively selected in the online survey platform. Participants could optionally provide their email address to enable a future follow-up survey.

Non-response and item-level missing data: Recruitment took place between November 2022 and May 2023, with two reminder emails sent to clinic chiefs. Item-level non-response was permitted throughout and not imputed, and individual questions could be skipped without invalidating the submission. Whole-survey non-response could not be assessed, as the total number of physicians to whom the survey was forwarded by department heads is unknown.

The study was approved by the Ethics Committee Leipzig, Germany (218/19-ek, 21 September 2021).

### 2.3. Statistical Analysis

All analyses were conducted as available-case analyses, with percentages and descriptive statistics calculated based on the number of respondents who answered each item. IBM SPSS Statistics (version 29.0, IBM Cooperation, Armonk, NY, USA) was used for all statistical analyses. The association between hospital level and participation in certificated courses or any in-house team training was assessed by binary logistic regression complemented by Pearson *χ*^2^ and Fisher–Freeman–Halton tests. Reporting follows the CROSS Checklist for Reporting of Survey Studies (Supplementary Table S1) [10].

## 3. Results

### 3.1. Participant Characteristics

**Figure 2:**
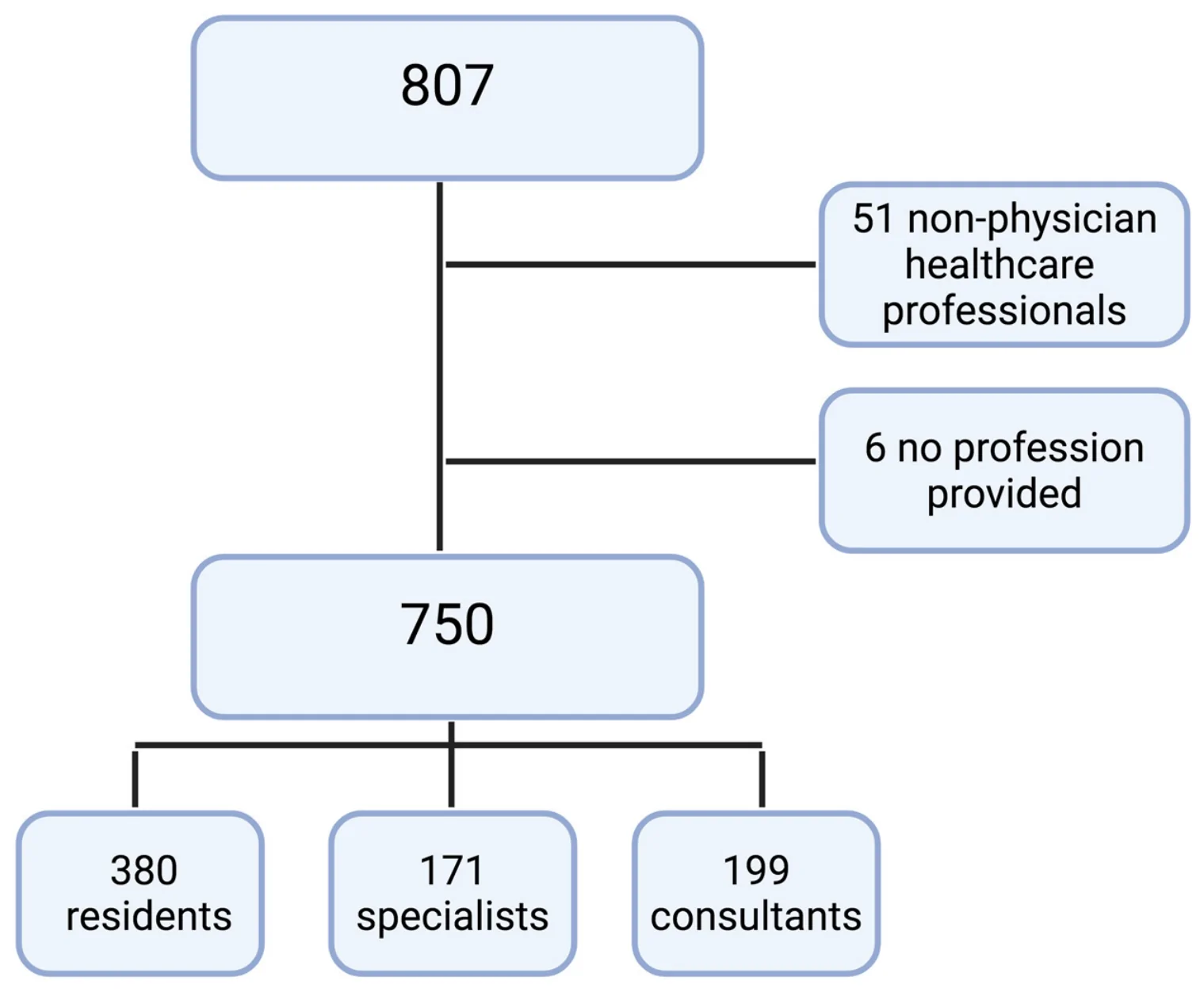
Flowchart of survey respondents (*N* = 807). After exclusion of non-paediatric specialists (*n* = 51) and respondents who did not provide their profession (=non-respondents; *n* = 6), a total of 750 participants were included in the analysis.

A total of 807 responses were received, 750 met the eligibility criteria and were included in the analysis. Of the excluded responses, 51 were from non-physician healthcare professionals (15 paramedics, 32 nurses, four others, six non-respondents), who were not included due to the small and heterogeneous sample size (Figure 2).

**Figure 3:**
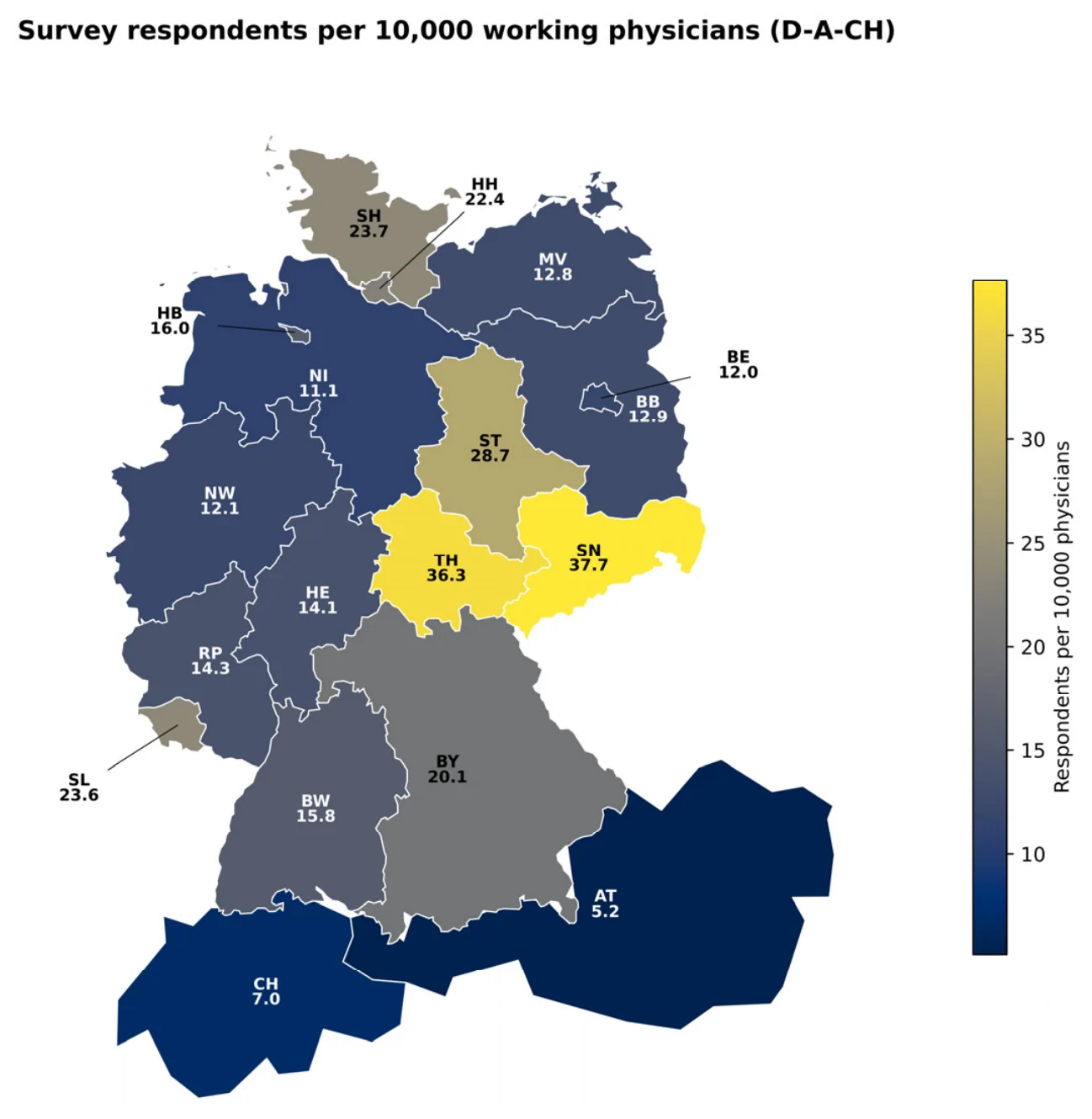
Geographic distribution of survey respondents (*N* = 805) per 10,000 physicians. BW = Baden-Wuertemberg, BY = Bavaria, BE = Berlin, BB = Brandenburg, HB = Bremen, HH = Hamburg, HE = Hesse, MV = Mecklenburg-Western Pomerania, NI = Lower Saxony, NW = North-Rhine Westphalia, RP = Rhineland-Palatinate, SL = Saarland, SN = Saxony, ST = Saxony-Anhalt, SH = Schleswig-Holstein, TH = Thuringia.

Of the eligible participants, 380 were paediatric residents with a median professional experience of 3 years, and 370 were specialists, senior physicians, or chief physicians (collectively referred to as senior paediatricians, SP) with a median work experience of 16 years. The majority of paediatric residents were female (72%); whereas, the SP group was evenly distributed (50% female). A comprehensive overview of respondent characteristics, workplace setting, and department affiliation is presented in Table 1. The geographic distribution of respondents is demonstrated in Figure 3 (and in detail in Supplementary Figure S1).

### 3.2. Implementation of Resuscitation Recommendations

**Figure 4:**
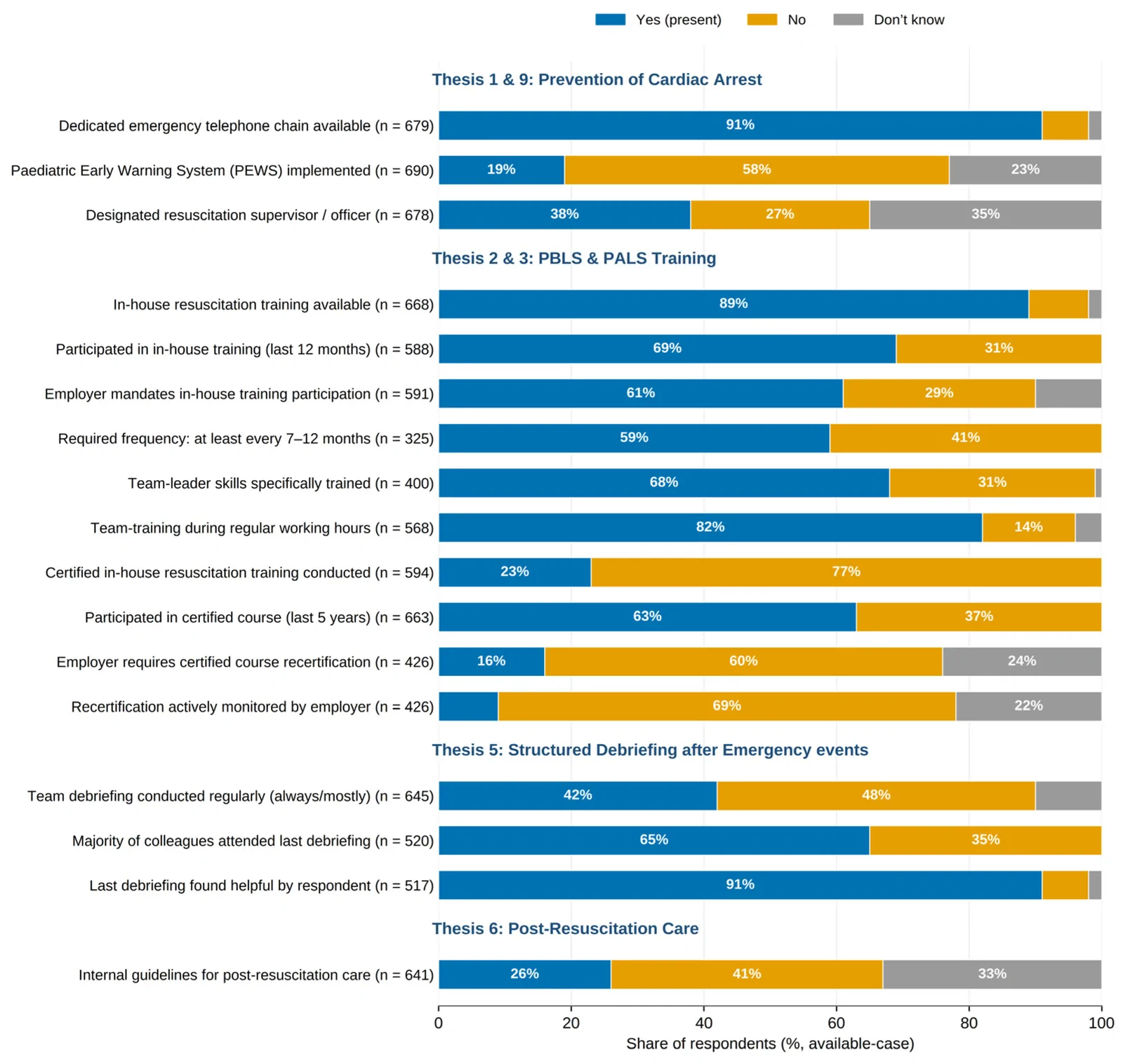
Proportion of respondents reporting implementation of each structural recommendation at their workplace. PEWS = Paediatric Early Warning System; PBLS = Paediatric Basic Life Support; PALS = Paediatric Advanced Life Support.

Figure 4 provides detailed numerical data of the implementation rate of the resuscitation recommendations. Seven respondents (<1%) reported that all recommendations were implemented at their institutions.

#### 3.2.1. Prevention of Cardiac Arrest

The majority of respondents reported the availability of a dedicated emergency telephone chain at their workplace (91%). Respondents were asked whether their workplace had “a paediatric early-warning score or another system to identify children whose state of health might deteriorate rapidly”; a PEWS or equivalent structured early-warning system was available at 19% of workplaces. Approximately one-third of participants indicated that a designated resuscitation supervisor was present at their institution (38%).

#### 3.2.2. PBLS and PALS Training

In-house resuscitation training was available at the workplace of 89% of respondents. 69% reported participation in in-house resuscitation training within the preceding 12 months. Training modalities were broadly distributed across case-based manikin training (347/594), team-based simulation training (344/594), and pure skills training (295/594). Targeted training in team-leader skills was received by 271/400 respondents.

The majority of employers required participation in in-house resuscitation training (61%). Of those specifying a frequency, the most common requirement was participation at least every 7–12 months (216/325, 66%). Certified in-house training was conducted by 23% of respondents with in-house training (137/594). Over one-third of respondents (248/663; 37%) reported not having participated in a certified resuscitation course in the previous 5 years. Employer-mandated recertification was required in 16% of cases, and compliance monitoring was reported in 9%.

#### 3.2.3. Structured Debriefing

Forty-two percent of the respondents reported that debriefings with the team following emergency events were conducted always or in most cases; whereas, 48% of the respondents indicated that debriefings were rarely or never conducted. Among those who had recently attended a debriefing, the majority (65%) reported that most colleagues had participated, and 91% found the session helpful.

#### 3.2.4. Post-Resuscitation Care

Internal procedural guidelines for paediatric post-resuscitation care were available at the workplace of 26% of respondents.

### 3.3. Comparison by Hospital Type

We compared the attendance for a certified PLS course over the last 5 years and for in-house training over the last 12 months at the hospital level. None of these criteria differed by hospital level (course: 57.1%/63.2%/62.4%, *χ*^2^ = 1.41, *p* = 0.49; in-house training: 66.1%/72.7%/65.7%, *χ*^2^ = 2.61, *p* = 0.27). Using general care hospitals as the reference, all odds ratios crossed unity (certified course: tertiary care OR 1.29, 95% CI 0.82–2.02; maximum/university care OR 1.24, 95% CI 0.83–1.87; in-house training: tertiary care OR 1.37, 95% CI 0.82–2.28; maximum/university care OR 0.98, 95% CI 0.63–1.54).

## 4. Discussion

The implementation of recommendations from current guidelines appears to be the major challenge of clinical medicine. Only seven individuals (<1%) reported working in a setting where all surveyed recommendations were in place. Taking into account that four of the ten recommended action plans were not surveyed, we assume that the implementing rate of all recommendations is even lower. Even though this survey does not allow us to determine whether the seven respondents are from one clinic or seven different clinics, it clearly demonstrates that the structural requirements for implementing paediatric resuscitation guidelines have not yet been adequately met across the D-A-CH region. In many cases, deficiencies were identified across both resource-intensive and less resource-intensive structural elements. Finally, while respondents from Saxony and Thuringia were somewhat over-represented, likely reflecting the regional professional networks of the coordinating authors, the survey captured participants from all German federal states as well as from Austria and Switzerland. Regional differences in training culture and institutional resources may introduce some heterogeneity, but the broad geographic reach of the survey supports its value as a comprehensive needs assessment across the D-A-CH region.

### 4.1. Prevention of Cardiac Arrest

Approximately one-third of respondents reported the presence of a designated resuscitation supervisor at their workplace. It is well established that explicit role assignment supports adherence to procedures, knowledge transfer, and adaptation of practice guidelines [11]. Although appointing a resuscitation coordinator at one’s own hospital does not incur additional costs or require significant material resources, the implementation of this action plan has so far been insufficient. One possible reason is that the team is expected to follow resuscitation guidelines even without a supervisor, or that the role is not considered particularly important.

A dedicated emergency telephone chain was available in the majority of workplaces, consistent with the ERC guideline recommendation for a clearly defined activation pathway. However, the guidelines provide no specification regarding the composition of the emergency team [12].

A PEWS or equivalent structured early-warning system was available to 19% of respondents. As the survey item was deliberately broad and encompassed any structured system serving this purpose rather than a specific validated PEWS model, the findings do not allow individual PEWS models to be distinguished. Allen et al. and Thomas-Jones et al. demonstrated in a prospective mixed-methods study that the requirements for implementing an early warning score vary considerably across hospital structures [13,14]. Both an effective afferent arm (rapid detection of the critically ill child) and an efferent arm (timely escalation of care) are required. Implementing a PEWS is complex and must be tailored to each hospital individually. Additional complicating factors include the costs (of training) and the extra time required, which adds to the daily workload. These findings underscore the absence of a universal approach to PEWS implementation and highlight the need for locally adapted, collaborative development processes.

### 4.2. Paediatric Basic and Advanced Life Support Training

While in-house resuscitation training was available to most respondents, 69% had participated in team training in the preceding 12 months. Although the majority of employees reported being able to attend training during working hours, the remaining 14% were required to use personal time. Contributing factors may include insufficient motivation, unclear learning objectives, work-related fatigue, concerns about embarrassment in simulation settings, or inaccurate self-assessment [7].

The content, frequency, and monitoring of training varied substantially. A total of 23% of respondents reported conducting certified in-house training. The reasons for this are likely multifactorial, including resource constraints (personnel, time, financial), and the absence of standardised guidelines for in-house team training outside the formal PBLS/PALS course structures offered by the ERC, which require considerable time and cost.

Targeted training in team leader skills was reported by 68% of those who received structured training. A large body of evidence highlights the central role of non-technical skills—including leadership, situational awareness, and communication—in the quality of emergency care [15].

Regular repetition is essential to maintain learned competencies [16]. Nevertheless, fewer than a third of respondents were required to attend in-house training at least once every 24 months, and 37% had not participated in any certified resuscitation course over a five-year period. Employer-mandated and monitored recertification was reported by 16% and 8% of respondents, respectively.

The recently published paediatric resuscitation guidelines explicitly address this gap, recommending clearly defined competency expectations for PBLS and PALS, as well as regular training incorporating team-based formats [1].

### 4.3. Structured Debriefing

Less than half of the respondents reported that debriefings following emergency events were conducted always or in most cases, while 48% indicated they were rarely or never conducted. This is consistent with other studies reporting similarly low rates of debriefings in paediatric emergency care [17,18]. This is a significant gap, given that those who did attend debriefings found them overwhelmingly beneficial (91%) and reported high colleague participation (65%). Cheng et al. have demonstrated that structured debriefing is associated with meaningful improvements in performance and skill acquisition [19]. The primary barrier to debriefing appears to be not reluctance, but time: creating the cultural and institutional space for reflection after resuscitation events requires sustained leadership commitment and protected time.

### 4.4. Post-Resuscitation Care

Internal procedural guidelines for post-resuscitation care were available in 26% of institutions. The absence of such guidelines does not necessarily imply that appropriate care is not being delivered; however, it suggests a lack of standardised, institution-level objectives for this critical phase. The current ERC guidelines allocate substantially greater attention to post-resuscitation management and, despite limited high-quality evidence, provide explicit targets to support structured care after cardiac arrest.

### 4.5. Regional and Institutional Comparisons

No significant differences in training participation rates were observed across hospital size or type. This finding suggests that implementation barriers are systemic rather than institution-specific, and that targeted national and supranational strategies are warranted.

## 5. Limitations

This study has several limitations. The online survey format did not technically prevent potential multiple submissions, and duplicate entries cannot be fully excluded. Distribution relied on clinic chiefs as gatekeepers, rather than direct contact with residents, introducing uncertainty regarding dissemination completeness.

The total number of paediatric residents and specialists in the D-A-CH region who received a survey invitation is unknown, precluding calculation of a response rate or formal assessment of sample representativeness. Due to the anonymous study design, it also cannot be established how many of the 327 German hospitals were actually reached. These factors introduce the possibility of both selection bias and volunteer bias: with physicians interested in paediatric resuscitation potentially over-represented, leading to overestimation of training uptake and institutional preparedness. Snowball sampling via professional networks may have further amplified this effect, consistent with the over-representation of respondents from Saxony and Thuringia relative to the regional physician workforce.

Several items showed notable non-response rates, possibly reflecting differential familiarity or uncertainty, and findings derived from these items should be interpreted with caution.

Furthermore, only six of the ten original theses were assessed in the survey; the four omitted theses partly concern administrative and structural features—a resuscitation registry, inter-hospital networking, and CPR-feedback systems—that individual respondents may be unable to report reliably; their omission may give an incomplete and potentially optimistic picture of regional preparedness. These methodological constraints should be considered when interpreting and generalising the findings.

## 6. Conclusions

The results of this survey clearly indicate that the structural prerequisites for implementing paediatric resuscitation recommendations have not been adequately met to date across the D-A-CH region. The reasons are likely multifactorial, and the observed implementation gaps are a cause for concern, as it is reasonable to speculate that insufficient structural preparedness may increase the risk of suboptimal resuscitation performance and of preventable harm in children following cardiac arrest.

Addressing this gap requires a coordinated effort across institutional, regional, and national levels. The integration of mandatory, certified PALS training into continuing medical education requirements for paediatric and adolescent medicine is strongly recommended. Furthermore, regular participation in both PBLS and PALS training should be mandated for all clinical staff, with employer monitoring of compliance. Supranational cooperation and quality improvement initiatives are essential to ensure that training programmes and the implementation of recommendations are adapted to the structural diversity of healthcare settings across Germany, Austria, and Switzerland.

It is time to invest the necessary financial and human resources to create optimal conditions for children to survive cardiac arrest with a favourable outcome. The cost of inaction—in terms of lives, quality of life, and long-term healthcare expenditure—is far greater than the cost of action.

## Figures and Tables

**Table 1:** Characteristics of survey respondents.

Characteristic	Total (*n* = 750)	Paediatric Residents (*n* = 380)	Senior Paediatricians (*n* = 370)
Gender			
Female	456/744 (61%)	271/377 (72%)	185/367 (50%)
Male	287/744 (38%)	106/377 (28%)	181/367 (49%)
Diverse	1/744 (<1%)	0/377 (0%)	1/367 (<1%)
Professional Experience			
Median years (IQR)	—	3 years (1.9–4)	16 years (10–20)
Country of Employment			
Germany	694/750 (92%)	368/380 (97%)	326/370 (88%)
Austria	27/750 (4%)	4/380 (1%)	23/370 (6%)
Switzerland	29/750 (4%)	8/380 (2%)	21/370 (6%)
Type of Hospital			
University hospital/maximum care	356/718 (50%)	167/368 (45%)	189/350 (54%)
General/district hospital	211/718 (29%)	117/368 (32%)	94/350 (27%)
County hospital	134/718 (19%)	83/368 (23%)	51/350 (14%)
Ambulatory healthcare centre/doctor’s office	17/718 (2%)	1/368 (<1%)	16/350 (5%)
No information	32	12	20
Primary Department [multiple answers]			
Doctor’s office/ambulatory healthcare centre/rehab/others	205/734 (28%)	76/369 (21%)	129/365 (35%)
Regular ward	242/734 (33%)	176/369 (48%)	66/365 (18%)
Intermediate paediatric care unit	106/734 (14%)	51/369 (14%)	55/365 (15%)
Paediatric/Neonatology intensive care	343/734 (47%)	161/369 (44%)	182/365 (50%)
Emergency room/delivery room [on-duty]	400/734 (55%)	242/369 (66%)	158/365 (43%)

IQR = interquartile range.

## Data Availability

The original contributions presented in this study are included in the article/Supplementary Materials. Further inquiries can be directed to the corresponding author.

## Supplementary Materials

The following supporting information can be downloaded at: https://www.mdpi.com/article/10.3390/children13080986/s1, Study Questionnaire File S1; Table S1: CROSS Checklist for Reporting of Survey Studies; Figure S1: Geographic distribution of respondents.
