# Supplementary material for: Implementation of Paediatric Life Support Recommendations: A Survey in the D-A-CH Region
Source: Children (Basel). 2026 Jul 24;13(8):986. doi: 10.3390/children13080986 (PMC13510385; doi:10.3390/children13080986)
Supplement: Supplementary file 1 [file children-13-00986-s001.zip › Study Questionnaire File S1.pdf]

Mandatory questions
Q1: Please state your clinical experience in years.
Q2: Please state your profession.<br>a) Pediatric resident<br>b) Pediatrician<br>c) Pediatric consultant/senior pediatrician<br>d) Other
Q3: Please state your biological sex.<br>a) female<br>b) male<br>c) non-binary<br>d) Other
Q7: Please state the federal state or country you are currently working in.<br>a) Baden-Württemberg<br>b) Bavaria<br>c) Berlin<br>d) Brandenburg<br>e) Bremen<br>f) Hamburg<br>g) Hessen<br>h) Mecklenburg-Western Pomerania<br>i) Lower Saxony<br>j) North Rhine-Westphalia<br>k) Rhineland-Palatinate<br>l) Saarland<br>m) Saxony<br>n) Saxony-Anhalt<br>q) Schleswig-Holstein<br>o) Thuringia<br>p) Austria<br>r) Switzerland
Q4: Please state the type of work you are currently working at.<br>a) Ambulatory healthcare center<br>b) county hospital<br>c) district hospital<br>d) tertiary university hospital<br>e) doctor´s office<br>f) other
Q5: In what departments are you working ?<br>a) doctor´s office<br>b) regular ward<br>c) ambulatory healthcare center<br>d) intermediate care unit<br>e) intensive care unit<br>f) regular ward and emergency department outside of normal work hours (nights, weekends)<br>g) regular ward and emergency department and birth clinic outside of normal work hours (nights, weekends)<br>h) emergency department<br>i) other

Q8: How many emergency situations with newborns, children or adolescents have you been involved in during the past 12 months?<br>a) none<br>b) 1-2<br>c) 3-5<br>d) 6-10<br>e) >10
Q8b: How many of these emergency situations did you have to manage as team leader?<br>a) none<br>b) 1-2<br>c) 3-5<br>d) 6-10<br>e) >10
Q8c: How many of these emergency situations did you have to manage as team member?<br>a) none<br>b) 1-2<br>c) 3-5<br>d) 6-10<br>e) >10
Q9: How many shifts do you have to work at the weekend or during the night/per month?<br>a) none<br>b) 1-2<br>c) 3-5<br>d) 6-10<br>e) >10
Q10: Does the hospital your are currently working in have an automated emergency call system in the event of an emergency?<br>a) Yes<br>b) No<br>c) I don´t know
Q11: Does the hospital your are currently working in have a person responsible for the implementation of resuscitation service and training?<br>a) Yes<br>b) No<br>c) I don´t know
Q11b: Does the hospital your are currently working in have an early warning system?<br>a) Yes<br>b) No<br>c) I don´t know
Q12: Does the hospital your are currently working in offer in-house resuscitation trainings?<br>a) Yes<br>b) No<br>c) I don´t know
Q13: Is this training mandatory?<br>a) Yes<br>b) No<br>c) I don´t know

Q15: In what frequency must this training be absolved?<br>a) at the begin of this employment<br>b) every 0-3 months<br>c) every 4-6 months<br>d) every 7-12 months<br>e) every 13-24 months<br>f) >every 2 years<br>g) other
Q14: Are the trainings absolved within your working hours?<br>a) Yes<br>b) No<br>c) I don´t know<br>d) Other
Q16: What type of training resembles your in-house training the most?<br>a) Skill training (e.g. focus on ventilation, compressions)<br>b) Simulations (with requirement of skills and focus on team-training including crew-resource management)<br>c) Case-based training of skills on manequins<br>d) Certified course formats (e.g. ERC PALS, PBLS, NLS, PILS)<br>e) Other certain course formats<br>f) Other
Q17: Did you attend an in-house training during the last twelve months?<br>a) Yes, once<br>b) Yes, twice<br>c) Yes, 3-5 times<br>d) Yes, 6-10 times<br>e) Yes, > 10 times<br>f) No
Q18: Did this course teach crew-resource-management elements?<br>a) Yes<br>b) No<br>c) Other
QO8: Did you absolve one or more of the following courses during the last five years?<br>a) EPALS<br>b) EPILS<br>c) NLS<br>d) PBLS<br>e) Paedsim<br>f) ETAT<br>g) No<br>h) PALS<br>i) Other
QO9: Does your employer foresee a recertification within the provided deadline?<br>a) Yes<br>b) No<br>c) I don´t know
QO10: Does your employer supervise the recertification?<br>a) Yes<br>b) No<br>c) I don´t know

QO11: Does your employer subsidise the participation of these courses financially?

a) Yes
b) No
c) Parts of it
d) I don´t know
e) Other

Q20: Does the hospital you currently work in offer regular debriefings after critical events?

a) No, never
b) Yes, only after a few events
c) Yes, after most of the events
d) Yes, after all of the events
e) I don´t know

Q20b: Are the debriefings mostly “hot” or “cold”?

a) Mostly hot, directly after the event
b) Mostly cold, some days after the event
c) We are doing both
d) Other
e)

Q21: Please specify the number of members of the involved staff who participated in the last debriefing you visited.

a) All of them
b) Most of them
c) Half of them
d) A few
e) None

Q22: Did you consider this last visited debriefing as helpful?

a) Yes
b) No
c) Other

Q23: Does your current work place have a structured post-resuscitation care plan?

a) Yes
b) No
c) I don´t know

Q24: To what extent do you feel adequately prepared to deal with paediatric emergency situations as team leader? (One indicating very low and five indicating very high)

a) 1
b) 2
c) 3
d) 4
e) 5

Q25: To what extent do you feel adequately prepared to deal with paediatric emergency situations as team member? (One indicating very low and five indicating very high)

a) 1
b) 2
c) 3
d) 4
e) 5

Q26: To what extent do you feel adequately trained in the utilization of the resuscitation algorithm? (One indicating very low and five indicating very high)

a) 1<br>b) 2<br>c) 3<br>d) 4<br>e) 5
Q27: To what extent do you feel adequately trained in communication during emergency events? (One indicating very low and five indicating very high)<br>a) 1<br>b) 2<br>c) 3<br>d) 4<br>e) 5
Q28: To what extent do you feel adequately trained in decision making during emergency events? (One indicating very low and five indicating very high)<br>a) 1<br>b) 2<br>c) 3<br>d) 4<br>e) 5
Q29: To what extent do you feel adequately trained in situational awareness during emergency events? (One indicating very low and five indicating very high)<br>a) 1<br>b) 2<br>c) 3<br>d) 4<br>e) 5
Q30: To what extent do you feel adequately trained in teamwork during emergency events? (One indicating very low and five indicating very high)<br>a) 1<br>b) 2<br>c) 3<br>d) 4<br>e) 5
Q31: Please indicate which training sessions you would like. In the comment fields, please<br>rank the desired training sessions, with 1 = most important, 2 = second most important, and so on.<br>a) I do not need further training<br>b) More observing within the clinical context/Video<br>c) More theoretical training<br>d) More simulation training<br>e) Team –leader training<br>f) In-house training: short skill training<br>g) In-house training: team training<br>h) Training within certified courses<br>i) other
**Optional questions**
Q03: What is the minimum time it takes for the on-call service to arrive at the patient's location after receiving the telephone notification?<br>a) consultant stays in the clinic, <2 minutes

b) 2-4 minutes
c) 5-10 minutes
d) >10-30 minutes
e) >30 minutes
f) other

QO4: What is the maximum time it takes for the on-call service to arrive at the patient's location after receiving the initial phone call?
a) consultant stays in the clinic, <2 minutes
b) 2-4 minutes
c) 5-10 minutes
d) >10-30 minutes
e) >30 minutes
f) other

QO5: How many people per instructor participated in the in-house team training session you last attended?
a) 1-3
b) 4-6
c) 7-10
d) >10
e) other

QO6: Do you receive feedback* as part of in-house CPR training (regardless of the training format)?
a) Yes, for all of the training
b) Yes, for most of the training
c) Yes, for some of the trainings
d) No, for no training

QO7: In what form will the evaluation take place?
a) Direct feedback from the resuscitation officer/trainer/instructor
b) Audio-visual (Video-feedback)
c) Objective feedback systems
d) other

QO12: How confident are you with mask-bag ventilation? (One indicating very low and five indicating very high)
a) 1
b) 2
c) 3
d) 4
e) 5

QO13: How confident are you in performing supraglottic airway management (e.g., laryngeal mask, pharyngeal (=Wendl) tube? (One indicating very low and five indicating very high)
a) 1
b) 2
c) 3
d) 4
e) 5

QO14: How confident are you with intubation? (One indicating very low and five indicating very high)
a) 1
b) 2

c) 3<br>d) 4<br>e) 5
QO15: How confident are you in setting up an IV line? (One indicating very low and five indicating very high)<br>a) 1<br>b) 2<br>c) 3<br>d) 4<br>e) 5
QO16: How confident are you in placing an intraosseous line? (One indicating very low and five indicating very high)<br>a) 1<br>b) 2<br>c) 3<br>d) 4<br>e) 5
QO17: How confident are you in determining the appropriate adrenaline dose? (One indicating very low and five indicating very high)<br>a) 1<br>b) 2<br>c) 3<br>d) 4<br>e) 5
QO18: How confident are you in performing CPR? (One indicating very low and five indicating very high)<br>a) 1<br>b) 2<br>c) 3<br>d) 4<br>e) 5
QO19: Mail-address for further surveys (optional)
